# SAT: Free Software for the Semi-Automated Analysis of Rodent Brain Sections With 2,3,5-Triphenyltetrazolium Chloride Staining

**DOI:** 10.3389/fnins.2019.00102

**Published:** 2019-02-12

**Authors:** Xiao-Fang Shi, Heng Ai, Wen Lu, Fuhong Cai

**Affiliations:** ^1^Department of Neurobiology, Key Laboratory of Medical Neurobiology of Ministry of Health of China, Zhejiang University School of Medicine, Hangzhou, China; ^2^Department of Physiology, Hangzhou Medical College, Hangzhou, China; ^3^Department of Biochemistry and Molecular Biology, Hainan Medical University, Haikou, China; ^4^Department of Electrical Engineering, Mechanical and Electrical Engineering College, Hainan University, Haikou, China

**Keywords:** ischemic stroke, middle cerebral artery occlusion, 2, 3, 5-triphenyltetrazolium chloride staining, semi-automated, algorithm

## Abstract

Ischemic stroke places an increasing burden on individuals, families, and societies around the world. However, effective therapies or drugs for ischemic stroke are lacking. Therefore, animal models mimicking ischemic stroke in humans are of great value for preclinical experiments. middle cerebral artery occlusion (MCAO) in mice or rats and subsequent 2,3,5-triphenyltetrazolium chloride (TTC) staining of brain sections are common methods in the study of experimental animal ischemic stroke. In this study, we present and assess the utility of the semi-automated analysis of the TTC staining (SAT) software program, a novel, small, user-friendly, and free software program, in the quantification of the infarct size in rodent brain sections, with TTC staining, by analyzing images captured by cell phones or scan systems. We performed MCAO and TTC staining in adult mice. We then utilized the SAT software and Image J to analyze the infarct size in the brain sections with TTC staining and compared the findings of the two analysis methods. We found that the data on infarct size from SAT and from Image J were comparable, suggesting that the SAT software could be an alternative option to Image J in the evaluation of ischemic stroke.

## Introduction

Stroke is the leading cause of death and disability worldwide (Feigin et al., 2014; Mozaffarian et al., 2016), and ischemic stroke is the most prevalent type of stroke (Moskowitz et al., 2010). Moreover, the prevalence of ischemic stroke has increased among adolescents and young adults (Mozaffarian et al., 2016). Thus far, the only effective therapy for ischemic stroke is recombinant tissue plasminogen activator (Chamorro et al., 2016; Khandelwal et al., 2016). However, this therapeutic approach is effective only within a critical time window (approximately 3–4 h after stroke occurs), and is limited (Moretti et al., 2015). Therefore, the development of novel drugs and approaches that extend the therapeutic window for ischemic stroke is crucial, and an extensive study is required.

Several rodent models of ischemic stroke have been developed and adopted for research (Woodruff et al., 2011). Middle cerebral artery occlusion (MCAO) in mice or rats is a popular model for mimicking focal ischemia in the brain (Liu and McCullough, 2011; Fluri et al., 2015). Following MCAO, the classic method utilizes 2,3,5-triphenyltetrazolium chloride (TTC) as a marker of ischemic damage to the brain tissue (Bederson et al., 1986; Türeyen et al., 2004). TTC reacts with intact mitochondrial oxidative enzyme systems and is subsequently reduced by the enzymes, which leads to the normal brain tissue being stained deep red in color (Straus et al., 1948; Bederson et al., 1986). In ischemic brain tissue, damaged mitochondria with impaired oxidative systems are incapable of reducing TTC (Bederson et al., 1986; Isayama et al., 1991); thus, the corresponding damaged brain tissue remains uncolored and easily distinguishable by the naked eye or algorithms. After experimental ischemic stroke modeling and TTC staining, accurate, and rapid quantification of the location and size of the infarct is critical for intervention selection. A widely accepted method for quantification of the infarct size in TTC staining utilizes the Image-Pro Plus or Image J, which requires software licenses or substantial manual operation.

In the current study, we present the semi-automated analysis of the TTC staining (SAT) software program, an open-source, small sized (approximately 18 KB), free to distribute and use software program (for Windows 7, Windows 8, and Windows 10), which provides semi-automated processing, analysis, and visualization of data of brain sections with TTC staining. We provide the developing principle and a detailed description of the user’s guide for the software. We also aim to assess the utility of the SAT program in the analysis of infarct sizes in rodent brain sections.

## Materials and Methods

### Animals

Eleven adult male C57/BL6 mice weighing 25–28 g were used in this study. The mice were group-housed at 23 ± 1°C with a 12-h light-dark cycle (lights on 08:00–20:00) and had free access to water and food. The surgical procedures and experimental protocols in this study were approved by Hangzhou Medical College and were in strict accordance to guidelines of the National Institutes of Health Guide for the Care and Use of Laboratory Animals. All efforts were made to minimize the suffering of the mice. The protocol was approved by Animal Advisory Committee of Hangzhou Medical College.

### MCAO and TTC Staining

Middle cerebral artery occlusion, which mimics focal cerebral ischemia, was induced in the right hemisphere as described previously (Zhang et al., 2013). Briefly, the mice were anesthetized by intraperitoneal injection of choral hydrate (350 mg/kg). Cerebral blood flow was monitored by laser Doppler flowmetry (Moor Instruments, Devon, United Kingdom). For transient MCAO, a 6-0 nylon monofilament (Cinontech Co., Ltd., Beijing, China) suture was gently inserted 10 mm into the internal carotid artery to occlude the origin of the middle cerebral artery. The suture was withdrawn smoothly to allow blood flow restoration after 60 min of occlusion. The body temperature was maintained at 37°C using a heat lamp (FHC, Bowdoinham, United States) during surgery and within 2 h after the onset of reperfusion.

At 24 h after surgery, the mice were anesthetized and decapitated, and the brains were removed and placed on ice. Coronal brain sections (2 mm thick) were stained with TTC (0.25%; Sigma-Aldrich, United States) at 37°C for 30 min in the dark, and post-fixed with formalin (4%).

### Infarct Analysis

For Image J analysis, the infarct size in each slice was traced and measured by manually outlining the margins of the non-ischemic areas using Image J 1.80^1^ (NIH, Bethesda, MD, United States). The infarct size was expressed as a percentage of the whole coronal section area. For SAT analysis, we followed the guidelines of the software described below.

#### Principle and Guidelines of SAT

The specimens were placed on a black background plate, and a camera or scan system was used to photograph the specimens. The digital image was loaded into our program and a mouse was used to select the target region. Because the brightness of the infarct area was greater than that of the normal tissue area and the black background plate, the brightness binarization method could be applied to extract the infarct area. However, the specimens and experimental conditions were unpredictable, so an adaptive brightness threshold value was needed to perform accurate binarization. In order to improve the applicability of our program, we provided a scrollbar (ScrBar1) in the program interface for users to alter the brightness threshold value, and this step requires manual operation. As we were dragging or clicking ScrBar1 to change the threshold value, the binarization results could be displayed on the interface in real time. This process of infarct area extraction was under users’ supervision. When the displayed result met the requirements of the user, a button (Set-Infarct) was pressed to proceed to the next step. Typically, this first process requires only a few seconds. We then needed to distinguish the normal area from the black background. Since the normal area presented as a red color, its red-channel brightness is greater than that of the background. Similar to the first step, a scrollbar (ScrBar2) was used to create a red-channel threshold value to extract the normal area from the background. We also applied the hue value to avoid interference of impurities in the specimens. The hue value of the normal area should be in the region of [-60 × F, 60 × F]. Herein, F represented a factor in the region of [0,0.2]. We could modify factor F using a third scrollbar (ScrBar3) to create a “digital red filter” to eliminate the interference of unexpected impurities. Typically, adjustment of ScrBar1 and ScrBar2 was sufficient for quantification of the infarct area. Generally, the above process requires <20 s if the user is familiar with the software. In the present study, the infarct size in the brain sections was determined by averaging the data obtained by two independent researchers.

### Data Analysis

All statistical graphs represent the mean ± standard error of the mean (SEM). Statistical analysis was conducted using a two-tailed paired Student’s *t*-test. Normal distribution was determined by the Kolmogorov-Smirnov test, and variance similarity was assessed by Fisher’s exact test. Correlation coefficient (R) and linear regression analysis were employed in order to compare our method and the conventional method for analysis of TTC staining. *P* ≤ 0.05 was considered to indicate significant differences.

### Software Availability

The SAT software and corresponding supplemental movie 1 in this study can be accessed at: http://dx.doi.org/10.17632/sbjnsv2shj.1

## Results

**FIGURE 1:**
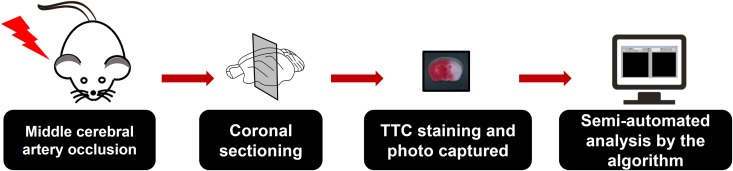
Overview of the semi-automated analysis of 2,3,5-triphenyltetrazolium chloride (TTC) staining software. Mice were subjected to middle cerebral artery occlusion (MCAO) and subsequent TTC staining. The coronal brain sections were then imaged under non-white and non-red backgrounds. Black backgrounds were preferred when using the semi-automated analysis of TTC staining (SAT) software for analysis. The images then underwent semi-automated analysis using the SAT software.

**FIGURE 2:**
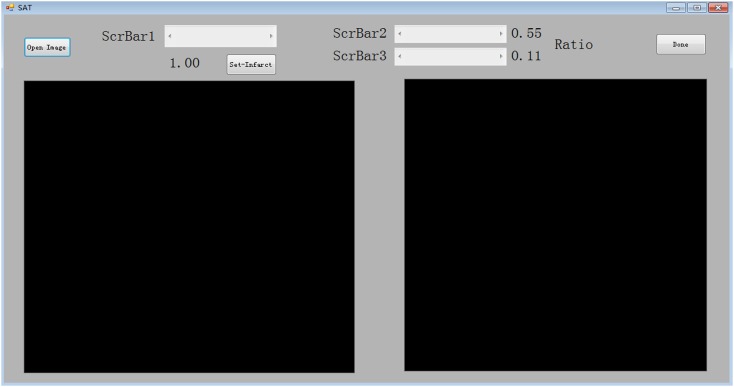
Image of the interface of the semi-automated analysis of TTC staining software.

**FIGURE 3:**
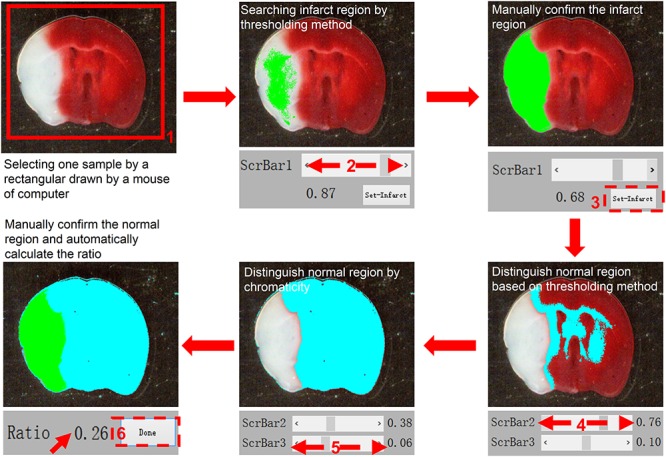
A standard workflow for analyzing images using the semi-automated analysis of TTC staining software. The TTC image file is first opened and loaded for analysis. In the first step, using a computer mouse to select the region of interest, the selected image is presented in the left panel. Following selection, “ScrBar1” is used for manual confirmation of the infarct area, and then “set-infarct” (inside the dashed line frame) is selected. Subsequently, “ScrBar2” is used for manual confirmation of the normal area. Occasionally, to avoid interference of impurities in the specimens, “ScrBar3” is used to eliminate the interference of unexpected impurities. Finally, the “Done” tab (inside the dashed line frame) is selected using the computer mouse. The data (Ratio) is presented in the upper right panel (indicated by red arrow here).

To facilitate the analysis of TTC staining of brain slices after MCAO and provide alternative choice for researchers, we sought out to develop a software, which is called SAT (semi-automated analysis for TTC staining). When utilizing the SAT, the MCAO model and TTC staining were conducted as usual, and the pictures of brain slices with TTC staining were captured under the specified condition (Figure 1, 2). To easily distinguish background and the brain slices by the algorithm, the background of the brain slices under capture should be non-white as well as non-red. Hence, we recommend that the user takes advantage of black plate as a background (Figure 1, 3). Since white background was used to present TTC staining figures in a large body of studies, it is worth to note this before calculation the infarct size by the SAT program. Following the protocols described in the Figure 3, the infarct size, which is expressed as a percentage of the whole brain slice, will be appeared in the software in real time (Figure 3). However, this software is not able to export the data as TXT format or Excel format after analysis at this time.

**FIGURE 4:**
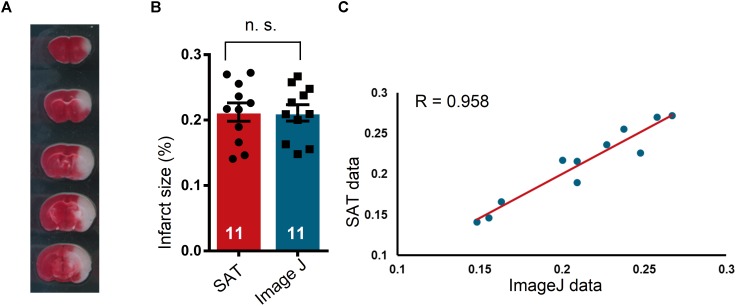
Comparison of semi-automated analysis of TTC staining software and Image J. **(A)** A representative TTC staining image for the subsequent analysis. **(B)** Comparison of the results of TTC-stained image analysis using semi-automated analysis of TTC staining (SAT) software and Image J. No significant differences were observed between the two analysis methods (*n* = 11 mice), n.s. indicates not significant. **(C)** Scatterplot of the SAT data and the Image J data. In the linear regression analysis of the results obtained by SAT and Image J analysis of brain sections with TTC staining (*n* = 11 mice), the regression coefficient, R, was 0.958.

In the present study, we performed MCAO and TTC staining to assess the accuracy and efficacy of SAT analysis of rodent brain sections by comparing the SAT results to the Image J results. We found that the SAT results were similar to the Image J results, and exhibited no significant differences (Figure 4A,B, *p* = 0.7721, *t* = 0.2976, df = 10). To further evaluate the utility of SAT analysis, we evaluated the relationship between these two methods. We observed a high level of correlation of the two data sets from the two methods (Figure 4C, *p* ≤ 0.0001, *R* = 0.958), suggesting that SAT analysis is comparable to Image J analysis.

## Discussion

In this study, we presented the protocol and guidelines for the use of our free software (SAT) for the analysis of brain sections with TTC staining. The development of the software was based on the brightness binarization method. Thus far, SAT has focused on the analysis of brain sections with TTC staining. In the near future, we hope it will be expanded to other applications, such as analyzing Nissl staining of brain sections after experimental stroke.

For the current version of SAT, we recognize that the data obtained by SAT were not able to export to TXT or Excel format. Moreover, analysis of TTC staining by SAT still requires manual supervision and operation. In addition, SAT can only function effectively when the image was captured on non-white and non-red backgrounds, but without the prerequisite of a specific photo capture system. SAT is capable of performing analysis with images captured by cell phones or scan systems. In addition, we used SAT to analyze several TTC-stained sections from previously published studies (see supplemental movie 1 for an example), thus suggesting the wide application of the software and indicating the quality of the previous published images as sufficient for analysis. In this study, we only applied SAT for analysis of TTC-stained mouse brain sections, but the software can also be applied to rat brain sections with TTC staining.

Recently, Macro in Image J has also been conducted in a semi-automated manner in the analysis of brain sections with TTC staining (Friedlander et al., 2017). SAT is not intended to replace Image J in the analysis of brain sections with TTC staining. However, we consider SAT to be a user-friendly and small sized software that provides an alternative analysis option for researchers in the field. Additionally, it benefits data reproducibility when the result is independently analyzed by two distinct methods.

In the future, we will aim to improve the SAT in four aspects. First, we will add the exporting function to the software, making the software capable of exporting data after analysis. Second, we are going to make the SAT able to concurrently analyze several brain sections, e.g., five sections, in a row or column. This parallel analysis may further reduce the time required and number of operational steps. Third, we are planning to develop this software as a smartphone application, making this algorithm easier to access and use. Fourth, we believe that deploying a deep learning algorithm, such as a convolutional neural network for the analysis, will improve our software in the future. By achieving the improvements mentioned above, we believe that the SAT will undoubtedly save user’s time, and result in the significant promotion of experimental studies on ischemic stroke.

## Author Contributions

X-FS, WL, and FC conceived the study. X-FS, HA, WL, and FC designed and analyzed the data. HA and WL performed the MCAO experiments and TTC staining. FC developed the software. X-FS, WL, and FC wrote the manuscript. All authors reviewed the results and approved the final version of the manuscript.

## Conflict of Interest Statement

The authors declare that the research was conducted in the absence of any commercial or financial relationships that could be construed as a potential conflict of interest.

## Footnotes

1 https://imagej.nih.gov/ij/
